# Fluoroquinolone Antibiotics Exhibit Low Antiviral Activity against SARS-CoV-2 and MERS-CoV

**DOI:** 10.3390/v13010008

**Published:** 2020-12-23

**Authors:** Stacey L. P. Scroggs, Danielle K. Offerdahl, Dylan P. Flather, Ciera N. Morris, Benjamin L. Kendall, Rebecca M. Broeckel, Paul A. Beare, Marshall E. Bloom

**Affiliations:** 1 Biology of Vector-Borne Viruses Section, Laboratory of Virology, Rocky Mountain Laboratories, National Institute for Allergy and Infectious Diseases, National Institutes of Health, Hamilton, MT 59840, USA; offerdahld@niaid.nih.gov (D.K.O.); dylan.flather@nih.gov (D.P.F.); ciera.morris@nih.gov (C.N.M.); kendall.benjamin@mayo.edu (B.L.K.); mbloom@niaid.nih.gov (M.E.B.); 2 Department of Molecular Medicine, Mayo Clinic College of Medicine, Rochester, MN 55905, USA; 3 Innate Immunity and Pathogenesis Section Laboratory of Virology, Rocky Mountain Laboratories, National Institute for Allergy and Infectious Diseases, National Institutes of Health, Hamilton, MT 59840, USA; rebecca.broeckel@nih.gov; 4 Coxiella Pathogenesis Section, Laboratory of Bacteriology, Rocky Mountain Laboratories, National Institute for Allergy and Infectious Diseases, National Institutes of Health, Hamilton, MT 59840, USA; pbeare@niaid.nih.gov

**Keywords:** SARS-CoV-2, Covid-19, MERS, fluoroquinolones, antiviral, efficacy, ciprofloxacin, enoxacin, levofloxacin, moxifloxacin

## Abstract

Repurposing FDA-approved drugs that treat respiratory infections caused by coronaviruses, such as SARS-CoV-2 and MERS-CoV, could quickly provide much needed antiviral therapies. In the current study, the potency and cellular toxicity of four fluoroquinolones (enoxacin, ciprofloxacin, levofloxacin, and moxifloxacin) were assessed in Vero cells and A549 cells engineered to overexpress ACE2, the SARS-CoV-2 entry receptor. All four fluoroquinolones suppressed SARS-CoV-2 replication at high micromolar concentrations in both cell types, with enoxacin demonstrating the lowest effective concentration 50 value (EC_50_) of 126.4 μM in Vero cells. Enoxacin also suppressed the replication of MERS-CoV-2 in Vero cells at high micromolar concentrations. Cellular toxicity of levofloxacin was not found in either cell type. In Vero cells, minimal toxicity was observed following treatment with ≥37.5 μM enoxacin and 600 μM ciprofloxacin. Toxicity in both cell types was detected after moxifloxacin treatment of ≥300 μM. In summary, these results suggest that the ability of fluoroquinolones to suppress SARS-CoV-2 and MERS-CoV replication in cultured cells is limited.

## 1. Introduction

Coronavirus disease 19 (COVID-19), severe acute respiratory syndrome (SARS), and Middle Eastern respiratory syndrome (MERS) are respiratory infections caused by viruses of the genus Betacoronavirus (family *Coronaviridae*)—SARS-CoV-2, SARS-CoV, and MERS-CoV, respectively [1,2]. Betacoronaviruses have clear epidemic and pandemic potential, as demonstrated by the emergence and transmission of SARS-CoV in 2002 [3], MERS-CoV in 2012 [4], and SARS-CoV-2 in 2019 [5]. The most pervasive and devastating of the three, SARS-CoV-2, has infected more than 77 million people and resulted in more than 1.7 million deaths worldwide as of December 2020 [6]. Effective and safe therapies are desperately needed to treat COVID-19 as well as other betacoronavirus infections that are likely to emerge in the future. A massive worldwide effort to identify antivirals has, thus far, resulted in the FDA approval of remdesivir to treat COVID-19 [7,8]. However, remdesivir is only approved for the treatment of hospitalized patients over the age of 12 years old and must be administered intravenously [7,9,10]. Due to these limitations, additional antiviral therapies are needed. Repurposing FDA-approved drugs can shorten the development time of a new antiviral therapy and can present a viable alternative to the identification of novel antiviral compounds.

Fluoroquinolones are broad-spectrum antibiotics [11] that have also demonstrated antiviral potency against multiple positive-sense, single-stranded RNA viruses, such as dengue virus [12,13], Zika virus [12,13,14], hepatitis C virus [15], and rhinovirus [16], but the mechanism of action has not been fully elucidated. In response to the COVID-19 pandemic, fluoroquinolones have been suggested as a potential repurposed therapy to treat infection with SARS-CoV-2 [17,18,19,20]. A large in vitro screen of FDA-approved drugs identified enoxacin and levofloxacin as more potent SARS-CoV-2 inhibitors compared to arbidol, a positive antiviral control [17]. Two in silico molecular docking studies identified enoxacin [18], ciprofloxacin, and moxifloxacin [19] as prospective SARS-CoV-2 inhibitors due to their predicted binding affinities to the SARS-CoV-2 main protease. Based on their pharmacokinetic properties, safety profiles, anti-inflammatory activity, and predicted binding affinities with the SARS-CoV-2 protease, levofloxacin and moxifloxacin have been proposed as treatments or co-treatments for COVID-19 pneumonia [20]. Because SARS-CoV-2 and MERS-CoV have single-stranded, positive-sense RNA genomes, we hypothesized that enoxacin, ciprofloxacin, levofloxacin, and moxifloxacin would suppress the replication of both betacoronaviruses in cell culture.

In this study, we found that, at high micromolar concentrations, multiple fluoroquinolones exhibit antiviral potency against SARS-CoV-2 in Vero and A549 cells that overexpress human ACE2 (A549/ACE2) and MERS-CoV in Vero cells. We detected low levels of cellular toxicity following treatment with the fluoroquinolones. Drug potency, measured by the effective concentration 50 (EC_50_), in the nanomolar range is an indicator of antiviral success [21]. The observed minimal antiviral potency of fluoroquinolones against SARS-CoV-2 and MERS-CoV in cell culture was not compelling enough to justify in vivo studies. However, it is possible that synergism with other drugs could improve the antiviral activity of fluoroquinolones to suppress the replication of betacoronaviruses.

## 2. Materials and Methods 

### 2.1. Cells

Vero (African green monkey kidney epithelial) cells were obtained from ATCC. Vero cells were maintained at 37 °C with 5% CO_2_ in Dulbecco’s minimum essential media (DMEM; Gibco, Thermo Fisher Scientific, Waltham, MA, USA) supplemented with 10% fetal bovine serum (FBS; Gibco, Thermo Fisher Scientific, Waltham, MA, USA) and 0.05 mg/mL of gentamycin (Gibco, Thermo Fisher Scientific, Waltham, MA, USA), hereafter referred to as complete DMEM. 

A549 (human lung epithelial) cells (ATCC CCL-185) were engineered to overexpress ACE2, the SARS-CoV-2 entry receptor. An ACE2 lentivirus was generated by transfecting 5 × 10^6^ HEK293T (human embryonic kidney) cells with 9 μg pWPI-Ace2 (Addgene plasmid #154981), 8 μg pSPAX2, and 1 μg pMD2.G using the ProFection Mammalian Transfection System (Promega, Madison, WI, USA). The media were changed, and lentiviruses were harvested 48 h after transfection and passed through a 0.45 μM filter. Low-passaged A549 cells were transduced with freshly prepared ACE2 lentivirus. Three days after transduction, A549 cells stably expressing ACE2, hereafter referred to as A549/ACE2, were selected with 5 μg/mL of blasticidin (Thermo Fisher Scientific, Waltham, MA, USA) for 3 days. A549/ACE2 cells were maintained at 37 °C with 5% CO_2_ in DMEM supplemented with 10% FBS and 5 μg/mL blasticidin. ACE2 expression in wild-type A549 and A549/ACE2 cells was validated via Western blot. Cells were lysed in RIPA buffer (25 mM Tris, 150 mM NaCl, 0.1% SDS, 0.5% sodium deoxycholate, and 1% Triton X-100) containing protease inhibitor (Roche, Basel, Switzerland) and protein was quantified with a Pierce BCA assay (Thermo Fisher Scientific, Waltham, MA, USA) [22]. Fifteen micrograms of lysate were loaded on a 4–12% BisTris NuPAGE gel (Invitrogen, Carlsbad, CA, USA) and SDS-PAGE was performed at 110 V for 2 h. Proteins were then transferred to a PVDF membrane via an iBlot gel-transfer device (Thermo Fisher Scientific, Waltham, MA, USA). The membrane was blocked with Odyssey TBS Blocking Buffer (LI-COR, Lincoln, NE, USA), then incubated overnight at 4 °C with the primary antibodies anti-ACE2 (ab15348; Abcam, Cambridge, UK) and anti-β-actin (8H10D10; Cell Signaling Technology, Danvers, MA, USA), followed by a 1 h, room temperature incubation with fluorescently labeled IRDye^®^ secondary antibodies (LI-COR, Lincoln, NE, USA). Membranes were visualized using an Odyssey CLx Imaging System (LI-COR, Lincoln, NE, USA). 

### 2.2. Viruses

SARS-CoV-2 (nCoV-WA1-2020 passaged three times in Vero cells, MN985325.1) [23] was obtained from the Centers for Disease Control and Prevention and subsequently passaged once in Vero E6 cells by Dr. Emmie de Wit [8]. For this study, the virus was additionally passaged once in Vero cells. MERS-CoV (HCoV-EMC/2012, passaged six times in Vero cells, NC_019843.3) was obtained from the Department of Viroscience, Erasmus Medical Center, Rotterdam, The Netherlands, and subsequently passaged once in Vero E6 cells by Dr. Emmie de Wit [24]. The virus was passaged once more in Vero cells in DMEM media supplemented with 2% FBS and 0.05 mg/mL gentamycin.

### 2.3. Virus Quantification

Viral titers were determined by a plaque assay [25]. One day before the plaque assay, 2 × 10^5^ Vero cells per well were plated onto tissue culture-treated 12-well plates. At the time of infection, samples were serially diluted in complete DMEM media then inoculated onto the Vero cells. After a 1 h incubation period at 37 °C with 5% CO_2_, the inoculum was removed and the wells were washed with 1x PBS followed by the addition of 1mL of 1.5% carboxymethylcellulose (Sigma-Aldrich, St. Louis, MO, USA) in OPTIMEM (Gibco, Thermo Fisher Scientific, Waltham, MA, USA). The plates were incubated for 96 h at 37 °C with 5% CO_2_ before fixation with 1 mL per well of 10% formalin (Cancer Diagnostics, Durham, NC, USA) for 1 h at room temperature. The formalin was removed, the wells were washed with 1× PBS (Phosphate-buffered saline; Gibco, Thermo Fisher Scientific, Waltham, MA, USA), and 250 μL per well of 0.1% crystal violet stain in ethanol was added. After 10 min incubation at room temperature, the stain was removed and the wells were washed twice with water. Viral plaques were counted to calculate viral titer as plaque forming units per mL (PFU/mL). SARS-CoV-2 replication in wild-type A549, A549/ACE2, and Vero cells was measured with a multicycle replication curve initiated at an MOI of 0.01. Cell supernatants were collected 24, 48, 72, and 96 h post-infection and quantified via plaque assay, as described above.

### 2.4. Antiviral Compounds

The fluoroquinolones evaluated in this study were enoxacin (Alfa Aesar, Tewksbury, MA, USA), ciprofloxacin (Sigma-Aldrich, St. Louis, MO, USA), levofloxacin (Sigma-Aldrich, St. Louis, MO, USA), and moxifloxacin (Sigma-Aldrich, St. Louis, MO, USA) (Figure S1). The drugs were prepared as previously described [12]. Briefly, the ciprofloxacin, levofloxacin, and moxifloxacin were solubilized in millipure water to 1.5 mM. Enoxacin was solubilized in ultrapure water to 1.5 mM with lactic acid (3 mM; Sigma-Aldrich, St. Louis, MO, USA) to improve the solubility. Following solubilization, all drugs were filter-sterilized with a 0.2 μm filter and diluted to 600 μM in the cell specific media before further 2-fold serial dilution in the cell specific media.

### 2.5. Determination of Effective Concentration 50 (EC_50_) for Fluoroquinolones against SARS-CoV-2 and MERS-CoV

The potency of enoxacin, ciprofloxacin, levofloxacin, and moxifloxacin was evaluated by calculating the EC_50_ values in Vero cells for SARS-CoV-2. The EC_50_ values for enoxacin were also determined for SARS-CoV-2 in A549/ACE2 cells and for MERS-CoV in Vero cells. Twenty-four hours before infection, 5 × 10^4^ cells per well were plated onto tissue culture-treated 24-well plates. Enoxacin, ciprofloxacin, levofloxacin, and moxifloxacin were solubilized as described above and diluted in complete DMEM to 600 μM before two-fold serial dilution in complete DMEM. Two negative control treatments were incorporated into these studies: complete DMEM alone and 150 μM lactic acid in complete DMEM. The known antiviral compound niclosamide was used as a positive control treatment (50 μM in DMEM) [26,27,28,29]. Triplicate wells were pretreated for 1 h with each drug at each concentration including the DMEM control. At the time of infection, the media or drug were removed and the wells were infected with SARS-CoV-2 or MERS-CoV at a MOI of 0.1 diluted in each drug concentration. After a 1 h incubation, the virus was removed, the cells were washed with 1x PBS, and the drug treatments were added to the wells. Viral supernatants were collected after 48 h of incubation at 37 °C with 5% CO_2,_ clarified by centrifugation, and stored at −80 °C. Viral titers were determined via plaque assay on Vero cells. The EC_50_ was calculated from the viral titers (log_10_PFU/mL) and the log drug concentrations in Prism (version 8; GraphPad Software, San Diego, CA, USA)) using a nonlinear regression model.

### 2.6. Anti-SARS-CoV-2 Activity of Fluoroquinolones in Human Lung Cells that Overexpress ACE2 Receptor

The efficacy of enoxacin, ciprofloxacin, levofloxacin, and moxifloxacin to suppress SARS-CoV-2 replication in A549/ACE2 cells was determined using three concentrations of the respective compound. Twenty-four hours before infection, 5 × 10^4^ cells per well were plated onto tissue culture-treated 24-well plates. Enoxacin, ciprofloxacin, levofloxacin, and moxifloxacin were solubilized as described above and diluted in complete DMEM to 600, 300, and 150 μM. The infection procedure and controls were the same as those described in Section 2.5. Viral titers were assessed for normality, then one-way ANOVAs in R (version 3.5.3) [30] with pairwise *t*-test comparisons were used to identify differences in mean viral titers of the drug treatments compared to the media control. 

### 2.7. Cellular Toxicity of Fluoroquinolones in Vero and A549 Cells

The fluoroquinolone toxicity for Vero and A549/ACE2 cells was evaluated based on the methods used to evaluate fluoroquinolone toxicity in human embryonic kidney cells (HEK-293) [12]. Briefly, 8 × 10^4^ cells per well were plated onto a tissue culture-treated 96-well plate, with one plate per cell type. To avoid drying at the edge of the plate, PBS only was added to the edge wells. After 24 h, media was removed from the wells that contained cells and 100 μL of drug or DMEM control was added to the wells, with three replicates per treatment. Two-fold serial dilutions of each drug ranged from 600 to 4.69 μM. The plates were incubated for 48 h at 37 °C with 5% CO_2_. After incubation, the treatments were removed from the wells and 100 μL of 10% alamarBlue™ cellular viability reagent (Invitrogen, Carlsbad, CA, USA) in complete DMEM per well was added to the wells. Plates were incubated at 37 °C with 5% CO_2_ for 2 h then the absorbance was measured via a plate reader at 570 λ. Normality was assessed for the raw absorbance values by Shapiro–Wilk tests. One-way ANOVA followed by pair-wise *t-*tests with Bonferroni correction in R (version 3.5.3) [30] was used to evaluate the differences in raw absorbance between the DMEM control and each treatment. The absorbance values for each cell type and drug concentration were normalized to the DMEM control by dividing each absorbance by the average absorbance of the DMEMs control to obtain the change in absorbance at each drug concentration per drug per cell type. The cytotoxic concentration 50 value (CC_50_) was calculated from the normalized absorbance values in Prism (version 8) using a nonlinear regression model. 

## 3. Results

### 3.1. High Micromolar Concentrations of Enoxacin Suppress SARS-CoV-2 and MERS-CoV Replication in Vero and A549/ACE2 Cells

The antiviral activity of enoxacin in SARS-CoV-2 infection was evaluated in Vero and A549/ACE2 cells. A549/ACE2 cells were utilized, as SARS-CoV-2 does not replicate in A549 cells that do not express ACE2 (Figure S2). The potency of enoxacin in suppressing MERS-CoV was also evaluated in Vero cells. Importantly, the cellular toxicity of enoxacin was also investigated. 

**Figure 1:**
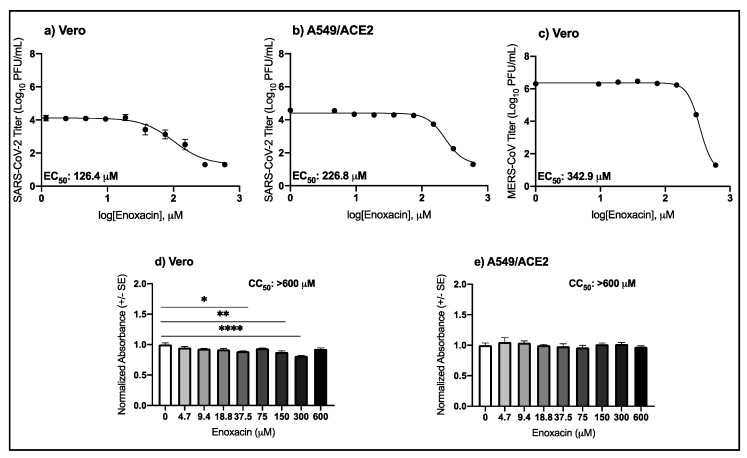
Antiviral activity of enoxacin against SARS-CoV-2 and MERS-CoV in cell culture. Mean SARS-CoV-2 titers (±SE) in (**a**) Vero and (**b**) A549/ACE2 cells. Mean MERS-CoV titers (±SE) in (**c**) Vero cells. Mean cellular viability in (**d**) Vero (one-way ANOVA; F (8,18) = 11.2, *p* = 1.4 × 10^−5^) and (**e**) A549/ACE2 cells (one-way ANOVA; F (8,18) = 0.7, *p* = 0.7). Pairwise *t-*tests from 0 μM: * *p* < 0.05, ** *p* < 0.001, **** *p* < 0.00001.

As shown in Table 1 and Figure 1a,b, enoxacin suppressed SARS-CoV-2 only at micromolar concentrations greater than 100 μM. The potency of enoxacin in Vero cells was slightly higher compared to the A549/ACE2 cells, as indicated by the lower EC_50_ value in the Vero cells, 126.4 μM compared to 226.8 μM in the human lung cells (Table 1). In both cell types, viral replication was below the level of detection following treatment with niclosamide, the positive antiviral control. MERS-CoV was also suppressed by enoxacin at high micromolar concentrations in Vero cells with an EC_50_ value of 324.9 μM (Figure 1c, Table 1). The Vero cellular viability after 37.5, 150, and 300 μM enoxacin was 10.9%, 12.2%, and 18.7% lower than the media control (Figure 1d; pairwise *t-*tests, *p* = 0.003, *p* = 0.0009, and *p* = 3.9 × 10^−6^, respectively), but the CC_50_ value was greater than 600 μM. The A549/ACE2 viability was not different from the media control for any concentration of enoxacin tested (Figure 1e). These results demonstrate that enoxacin suppresses SARS-CoV-2 and MERS-CoV at high micromolar concentrations with minimal or no cellular toxicity in Vero and A549/ACE2 cells, respectively.

### 3.2. High Micromolar Concentrations of Ciprofloxacin Suppress SARS-CoV-2 Replication 

**Figure 2:**
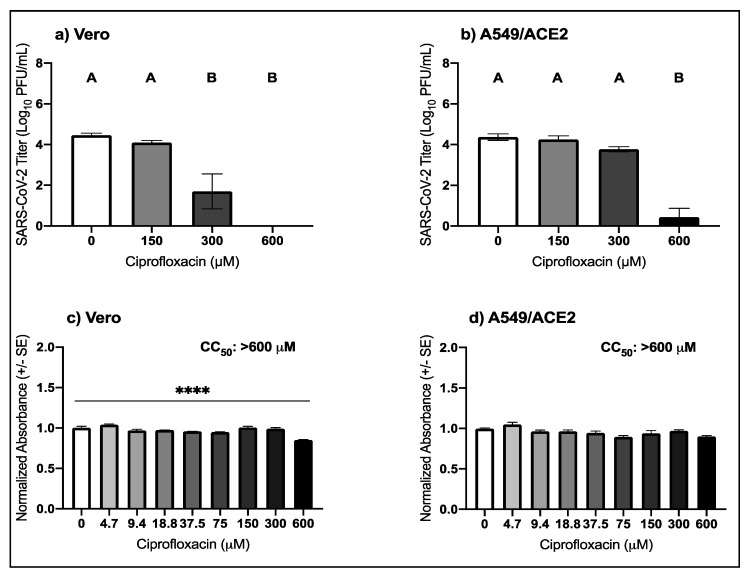
Antiviral activity of ciprofloxacin against SARS-CoV-2 in cell culture. Mean SARS-CoV-2 titers (±SE) in (**a**) Vero (one-way ANOVA, F (3,8) = 23.1, *p* = 0.0003) and (**b**) A549/ACE2 cells (one-way ANOVA, F (3,8) = 53.3, *p* = 1.3 × 10^−5^). Mean cellular viability in (**c**) Vero (one-way ANOVA; F (8,18) = 21.1, *p* = 1.2 × 10^−7^) and (**d**) A549/ACE2 cells (one-way ANOVA; F (8,18) = 5.6, *p* = 0.001). Values that do not share a letter are significantly different (*p* < 0.05). Pairwise *t-*tests from 0 μM: **** *p* < 0.00001.

The anti-SARS-CoV-2 activity and cellular toxicity of ciprofloxacin was evaluated in Vero and A549/ACE2 cells. In Vero cells, ciprofloxacin suppressed SARS-CoV-2 with an EC_50_ value of 246.9 μM (Table 1). The mean SARS-CoV-2 titer in Vero cells following 300 μM ciprofloxacin was 1.7 log lower than the control (pairwise *t-*test, *p* = 0.008) and below the level of detection after 600 μM treatment (Figure 2a). In the A549/ACE2 cells, the mean SARS-CoV-2 titer was decreased by 3.9-log following treatment with 600 μM of ciprofloxacin (Figure 2b; pairwise *t-*test, *p* = 2.7 × 10^−5^). The SARS-CoV-2 titer was below the level of detection following treatment with the positive antiviral control, niclosamide. In Vero cells, there was a 14.8% decrease in the mean normalized absorbance compared to the control following 600 μM ciprofloxacin treatment (Figure 2c, pairwise *t-*test, *p* = 9.5 × 10^−7^); however, the CC_50_ value was greater than 600 μM. Ciprofloxacin treatment at any of the concentrations tested did not impact the cellular viability of A549/ACE2 cells (Figure 2d). These results indicate that ciprofloxacin suppresses SARS-CoV-2 replication with little to no cellular toxicity.

### 3.3. High Micromolar Concentrations of Levofloxacin Suppress SARS-CoV-2 Replication 

**Figure 3:**
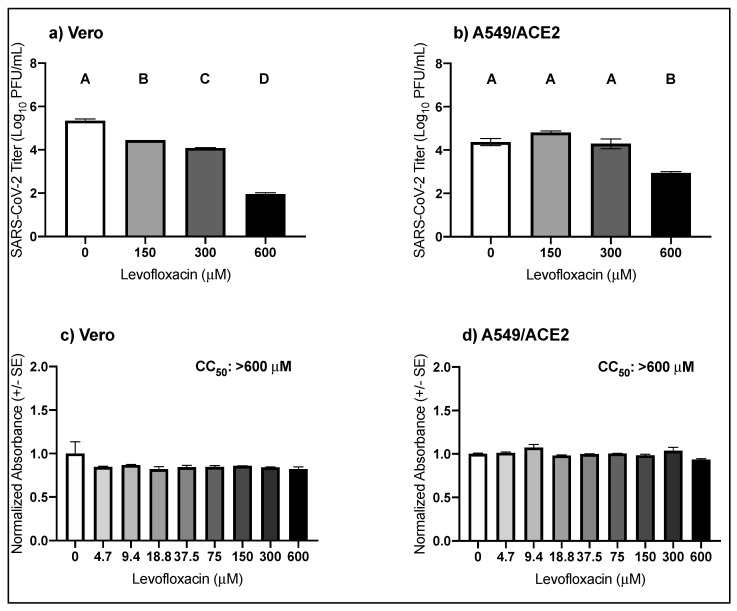
Antiviral activity of levofloxacin against SARS-CoV-2 in cell culture. Mean SARS-CoV-2 titers (+/- SE) in (**a**) Vero (one-way ANOVA, F (3,8) = 786.0, *p* = 3.2 × 10^−10^) and (**b**) A549/ACE2 cells (one-way ANOVA, F (3,8) = 30.5, *p* = 9.9 × 10^−5^). Mean cellular viability in (**c**) Vero (one-way ANOVA; F (8,18) = 1.3, *p* = 0.3) and (**d**) A549/ACE2 cells (one-way ANOVA; F (8,18) = 3.8, *p* = 0.009). Values that do not share a letter are significantly different (*p* < 0.05).

The anti-SARS-CoV-2 activity and cellular toxicity of levofloxacin were evaluated in Vero and A549/ACE2 cells. Treatment with increasing concentrations of levofloxacin in Vero cells reduced SARS-CoV-2 viral titers by almost 1-log for 150 μM, 1.3-log for 300 μM, and 3.4-log for 600 μM (Figure 3a; pairwise *t-*tests, *p* = 3.2 × 10^−6^, *p* = 3.5 × 10^−7^, and *p*= 2.9 × 10^−10^, respectively) with an observed EC_50_ of 418.6 μM (Table 1). In the A549/ACE2 cells, the SARS-CoV-2 titer was decreased by 1.4-log following treatment with 600 μM of levofloxacin (Figure 3b; pairwise *t-*test, *p* = 0.0006). For all concentrations tested, the cellular viability compared to the media control was unaffected by levofloxacin treatment, regardless of cell type (Figure 3c,d). These results suggest that levofloxacin moderately suppresses SARS-CoV-2 replication without detectable toxicity.

### 3.4. High Micromolar Concentrations of Moxifloxacin Suppresses SARS-CoV-2 Replication 

**Figure 4:**
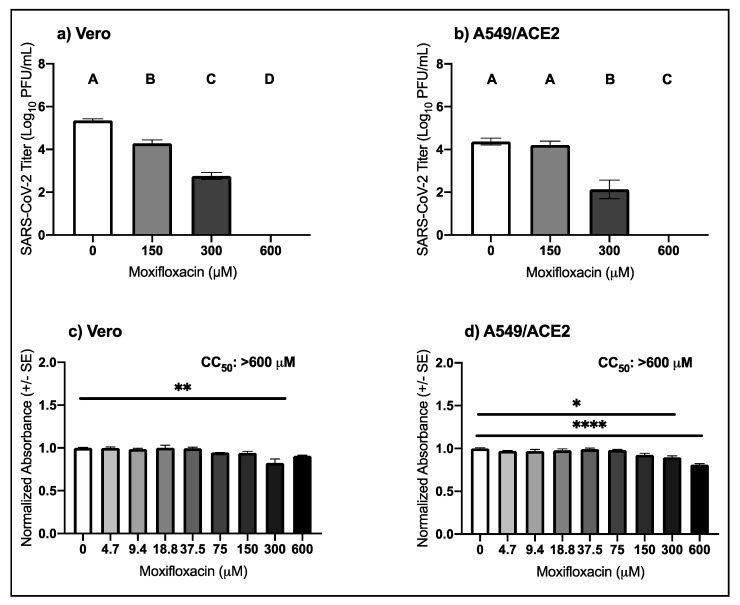
Antiviral activity of moxifloxacin against SARS-CoV-2 in cell culture. Mean SARS-CoV-2 titers (±SE) in (**a**) Vero (one-way ANOVA, F (3,8) = 377.6, *p* = 5.9 × 10^−9^) and (**b**) A549/ACE2 cells (one-way ANOVA, F (3,8) = 68.6, *p* = 4.8 × 10^−6^). Mean cellular viability in (**c**) Vero (one-way ANOVA; F (8,18) = 8.1, *p* = 0.0001) and (**d**) A549/ACE2 cells (one-way ANOVA, F (8,18) = 15.9, *p* = 1.1 × 10^−6^). Values that do not share a letter are significantly different (*p* < 0.05). Pairwise *t-*tests from 0 μM: * *p* < 0.05, ** *p* < 0.001, **** *p* < 0.00001.

The anti-SARS-CoV-2 activity and cellular toxicity of moxifloxacin was evaluated in Vero and A549/ACE2 cells. Increasing concentrations of moxifloxacin in both cell types resulted in a dose–response reduction in SARS-CoV-2 titer (Figure 4a,b). In Vero cells, SARS-CoV-2 titer was decreased by 1.1-log and 2.6-log following treatment with 150 μM and 300 μM moxifloxacin (pairwise *t-*tests, 150 μM *p* = 0.0002, 300 μM *p* = 9.8 × 10^−7^) with an observed EC_50_ of 239.7 μM (Table 1). Treatment with 600 μM moxifloxacin reduced SARS-CoV-2 titer to below the level of detection (pairwise *t-*tests, *p* = 6.5 × 10^−9^). In A549/ACE2 cells SARS-CoV-2 titer was decreased by 2.2-log following treatment with 300 μM of moxifloxacin (pairwise *t-*test, *p* = 0.009), and following 600 μM moxifloxacin the viral titers were below the level of detection (Figure 4b). Like the other fluoroquinolones, the CC_50_ values for moxifloxacin in both cell types were greater than 600 μM. However, after treatment of 300 μM moxifloxacin in Vero cells there was a 9.3% decrease in mean normalized absorbance compared to the control (Figure 4c; pairwise *t-*test, *p* = 0.0005). In A549/ACE2 cells, the mean normalized absorbance decreased by 10.4% (pairwise *t-*test, *p* = 0.004) and by 18.9% (pairwise *t-*test, *p* = 2.3 × 10^−6^) following treatment with 300 μM and 600 μM moxifloxacin (Figure 4d). These data indicate that moxifloxacin suppresses SARS-CoV-2 replication with minimal cellular toxicity. 

## 4. Discussion

Fluoroquinolones are known to suppress the replication of RNA viruses such as dengue, Zika, and hepatitis C viruses [12,13,14,15,16]. The antiviral mechanisms of action are not fully understood, but have been suggested to include interference with viral entry and the inhibition of the viral helicase [12,13,15]. Several fluoroquinolones have been identified in large antiviral screens or molecular docking analyses as possibly interfering with the SARS-CoV-2 infectious cycle. Enoxacin and levofloxacin were identified in an in vitro screen as potential SARS-CoV-2 inhibitors with activity in the low micromolar range [17], while ciprofloxacin was predicted to interact with the SARS-CoV-2 main protease using in silico models [18,19]. Based on similarities in structure, activity, and safety profiles, moxifloxacin and levofloxacin were proposed as potential treatments or adjuncts to attenuate SARS-CoV-2 respiratory symptoms [20]. As these drugs are FDA-approved, repurposing them as an antiviral therapy to combat the ongoing COVID-19 pandemic is an attractive option. We evaluated the potency of enoxacin, ciprofloxacin, levofloxacin, and moxifloxacin to suppress SARS-CoV-2 and MERS-CoV in two cell types and found antiviral activity only at high micromolar concentrations. The cell types used in this study were Vero cells, African green monkey kidney cells that are interferon-deficient [31,32], and A549 human lung cells that have been engineered to overexpress the SARS-CoV-2 entry receptor ACE2. As has been documented previously, fluoroquinolone antiviral potency likely varies by cell type and level of cellular differentiation [12,14]. 

The potency at high micromolar concentrations was consistent across fluoroquinolones, cell types, and viruses, but cellular toxicity varied by fluoroquinolone and cell type. While the CC_50_ values for all four drugs in both cell types were higher than 600 μM, moxifloxacin significantly impacted the cell viability of Vero and A549/ACE2 cells at 300 and 600 μM. Similarly, 600 μM ciprofloxacin negatively impacted the Vero cell viability. The decrease in viability for both drugs ranged from 9.3% to 18.9%. The decrease in viral titer following high micromolar ciprofloxacin and moxifloxacin treatment could be an indirect effect of the drugs’ impact on cell viability. However, the 4-log decrease in viral titer is much greater in magnitude than the 20% decrease in cell viability, indicating that the decrease in released virus at these concentrations of moxifloxacin and ciprofloxacin is due only in part to cellular toxicity. The high micromolar CC_50_ values are similar to what has been documented in human embryonic kidney cells (HEK-293), another human cell line [12]. Importantly, acute treatment in humans with enoxacin, ciprofloxacin, levofloxacin, or moxifloxacin is well tolerated in the general population, and severe side effects occur but are uncommon [33]. The EC_50_ values observed in this study were greater than 100 μM, in contrast to the low micromolar EC_50′_s documented for other RNA viruses [12,14,15]. On average, the betacoronavirus EC_50_ values are 16.5-fold higher for enoxacin and 7.0-fold higher for ciprofloxacin compared to the flavivirus and hepatitis C virus EC_50_ values [12,14,15]. The lowest EC_50_ we identified, 126.4 μM, was that of enoxacin in Vero cells against SARS-CoV-2, but enoxacin also demonstrated cellular toxicity at 37.5, 150, and 300 μM meaning that toxicity likely played a role in the decrease in viral titer. The EC_50_ values observed in this study were well above the peak serum concentrations that have been documented in humans as antibiotics [34,35,36,37,38,39]. The peak human serum concentrations of enoxacin, ciprofloxacin, and moxifloxacin following a typical dose of 400 to 600 mg are 11.2, 7.2, and 8.6 μM, respectively [35,37,38,39]. Together with our results, the data indicate that administering a typical fluoroquinolone dosage would not yield the serum concentration required to suppress 50% of virus replication.

Structural modifications may increase the antiviral utility of fluoroquinolones in regard to coronavirus treatment. Fluoroquinolones are a class of more than 20 compounds that share a 4-quinolone backbone with an attached fluorine that are grouped into four generations (Figure S1 [11]). Each fluoroquinolone has a unique composition of R groups, but some R groups are shared across multiple fluoroquinolones. For example, many fluoroquinolones contain a cyclopropane attached to the nitrogen of the 4-quinolone backbone. Levofloxacin contains a morpholine ring attached to the 4-quinolone backbone in contrast to ciprofloxacin and moxifloxacin, which contain a cyclopropane or enoxacin which contains an ethyl group at the same position. Moreover, the only difference between the chemical structures of levofloxacin and ciprofloxacin is the morpholine ring in place of the cyclopropane. As the EC_50_ value for levofloxacin was almost double that of ciprofloxacin, we hypothesize that the morpholine ring diminished the potency of levofloxacin. Future studies to evaluate a full panel of fluoroquinolones could narrow down which structural elements are important for antiviral activity and provide a starting point for structural manipulations to enhance efficacy. Of the four fluoroquinolones tested in this study, enoxacin was the most potent inhibitor, making it an obvious choice for further biochemical development. 

Successful antivirals often have EC_50_ values in the low nanomolar range [21]. While our data from cell culture support our hypothesis that fluoroquinolones suppress betacoronaviruses, the high micromolar EC_50′_s do not support the further evaluation of these drugs as direct suppressors of SARS-CoV-2 or MERS-CoV replication in animal models. However, fluoroquinolones attenuate the pro-inflammatory immune response in vivo, a trend that is not always consistent in cell culture (reviewed [40]). In severe cases of SARS [41], MERS [42], and COVID-19 [43], the secretion of a number of pro-inflammatory cytokines, including IL-6, IL-12, IFN-γ, and TNF-α, was increased compared to mild cases, suggesting that an overactive cytokine response plays a role in disease severity. The ability of fluoroquinolones to attenuate the overactive cytokine response warrants further study in animal models. Fluoroquinolones show some promise as a treatment for several viral infections, but the current study indicates that fluoroquinolone treatment alone is unlikely to have measurable clinical value against betacoronavirus infection.

In summary, we evaluated the antiviral potency and cellular toxicity of four fluoroquinolones against two betacoronaviruses in two cell types. We detected variable but minimal cellular toxicity following treatment with the fluoroquinolones, and while all four drugs suppressed betacoronavirus replication, the observed level of potency was low. Our results indicate that enoxacin, ciprofloxacin, levofloxacin, and moxifloxacin are not ideal antiviral candidates for the treatment of COVID-19 or MERS.

## Figures and Tables

**Table 1:** Effective concentration 50 values for enoxacin, ciprofloxacin, levofloxacin, and moxifloxacin in Vero and A549/ACE2 cells against SARS-CoV-2 and MERS-CoV.

Virus	Drug	Cell Type	EC_50_ μM (95% CI)
SARS-CoV-2	Enoxacin	Vero	126.4 (88.3–256.4)
	Enoxacin	A549/ACE2	226.8 (210.1–244.6)
	Ciprofloxacin	Vero	246.9 (197.6–313.3)
	Levofloxacin	Vero	418.6 (365.5–480.6)
	Moxifloxacin	Vero	239.7 (213.7–267.4)
MERS-CoV	Enoxacin	Vero	342.9 (324.0–368.5)

## Data Availability

The data presented in this study are available in Data S1.

## Supplementary Materials

The following are available online at https://www.mdpi.com/1999-4915/13/1/8/s1: Figure S1: Chemical structures of (a) enoxacin, (b) ciprofloxacin, (c) levofloxacin, and (d) moxifloxacin with 4-quinolone backbone highlighted in green. Colored boxes indicate structure of ethyl R group (Orange), cyclopropane R group (Purple), and morpholine R group (Blue). Figure S2: (a) Expression of human ACE2 protein in wild-type A549 cells and A549/ACE2 cells. (b) SARS-CoV-2 replication in wild-type A459, A549/ACE2, and Vero cells at 24 (grey), 48 (orange), 72 (blue), and 96 (pink) hours post infection (MOI: 0.1). Data S1: Excel spreadsheet containing the underlying data for Figure 1, Figure 2, Figure 3 and Figure 4 and Table 1.
